# Predictive modeling for survival-related outcomes in lung cancer patients with brain metastases: a mini-review

**DOI:** 10.3389/fonc.2026.1859715

**Published:** 2026-07-14

**Authors:** Sifat Jahan Shorna, Sreya Majumder, Diya Rahman, Fariha Jahan, Sheak Rashed Haider Noori, Liew Tze Hui, Dip Nandi, Mashiour Rahman

**Affiliations:** 1 Department of Computer Science and Engineering, Shahjalal University of Science and Technology (SUST), Sylhet, Bangladesh; 2 ELITE Research Lab, New York, NY, United States; 3 Department of Computer Science and Engineering, BRAC University (BRACU), Dhaka, Bangladesh; 4 Department of Computer Science, American International University-Bangladesh, Dhaka, Bangladesh; 5 Health Informatics Research Lab (HIRL), Department of Computer Science & Engineering, Daffodil International University, Dhaka, Bangladesh; 6 Faculty of Information Science and Technology, Multimedia University, Melaka, Malaysia

**Keywords:** brain metastases, deep learning, lung cancer, machine learning, multimodal learning, survival prediction

## Abstract

Predicting survival in lung cancer patients, particularly those with brain metastases, is crucial for personalizing treatment plans and estimating patient prognosis. This minireview synthesizes findings from fifteen recent studies published between 2020 and 2026, focusing on survival prediction in lung cancer patients with brain metastases, with key outcomes including overall survival, progression-free survival, and intracranial progressionfree survival. Traditional and current approaches for predicting survival are discussed, along with comparisons between unimodal and multimodal approaches. Moreover, the strengths and limitations of existing methods are critically analyzed. The findings emphasize the potential of advanced predictive modeling to inform personalized treatment plans and improve survival outcomes in this high-risk population.

## 1 Introduction

Brain metastases (BM) are a critical complication in lung cancer and occur in approximately 30-40% of patients (1). Prognosis for these patients is highly heterogeneous, influenced by a complex interplay of clinical, radiologic, pathologic, and molecular factors (2). Therefore, reliable estimation of survival at the time of diagnosis is important to guide treatment planning and improve patient management. Traditionally, survival prediction in lung cancer with brain metastases (LCBM) patients has been based on clinical scoring systems developed from statistical methods. In recent years, machine learning (ML) and deep learning (DL) methods have made it possible to extract detailed imaging features from CT, MRI, and PET scans. This has expanded survival modeling beyond basic clinical information. Recent advances in artificial intelligence (AI) and multimodal DL provide opportunities to integrate clinical and radiologic data. Building on this, this mini-review aims to address the following three key questions:

- RQ1: What are the traditional approaches in predicting survival in LCBM patients?
- RQ2: What are some existing models for predicting survival in LCBM patients?
- RQ3: Does multimodal data integration improve survival prediction performance compared to unimodal models?

## 2 Methodology of literature section

To identify the relevant studies, we performed a focused search across ScienceDirect, Springer Nature Link, IEEE Xplore, PubMed, Google Scholar, ResearchGate, and Elsevier. The final search was performed in February 2026, and studies published between October 2020 and February 2026 were considered eligible for review. Representative search strings included combinations of: “Lung Cancer with Brain Metastases” AND (“Graded Prognostic Assessment (GPA)” OR “Lung-molGPA” OR “Prognostic Factors” OR “Survival Prediction” OR “Machine Learning” OR “Deep Learning” OR “Artificial Intelligence”). Studies were included if they: (i) focused on LCBM patients; (ii) investigated survival prediction or prognostic scoring systems; (iii) used traditional statistical approaches or ML/AI methods; and (iv) were published as peer-reviewed journal articles in English. Studies were excluded if they: (i) did not specifically analyze LCBM patients; (ii) focused solely on diagnosis or treatment response rather than survival-related outcomes; (iii) were conference abstracts, editorials, case reports, or non-peer-reviewed publications; and (iv) were not available in English.

After evaluating titles, abstracts, and full texts for relevance and methodological quality, studies that most directly addressed prognostic assessment and survival prediction in LCBM were included. From this search, a total of fifteen studies were selected.

## 3 Discussion

We analyzed the selected primary research papers to extract information relevant to our research objectives and address the research questions.

### 3.1 RQ1: What are the traditional approaches in predicting survival in LCBM patients?

Among traditional prognostic scoring systems for predicting survival in LCBM patients, the Graded Prognostic Assessment (GPA) is widely used because of its simplicity and cost-effectiveness. A multi-institutional study (2) established the first GPA for small cell lung cancer (SCLC) and updated the Lung GPA of both SCLC and non-small cell lung cancer (NSCLC) patients. The median overall survival (OS) for NSCLC adenocarcinoma, SCLC, and NSCLC non-adenocarcinoma with BM was 17, 10, and 8 months, respectively. Study (3) evaluated the prognostic value of GPA scores in routine practice, where patients were stratified into GPA groups (0-1, 1.5-2, 2.5-3) and OS was 8 months. Similarly, Schröder et al. (4) calculated DS-GPA for NSCLC, lung-molGPA and GPA for NSCLC adenocarcinoma and reported OS values of 7 months for GPA 0-1, 16 months for GPA 1.5-2, 33 months for GPA 2.5-3, and not reached for GPA 3.5-4 (p *<* 0.001). In another study (5), the authors identified the prognostic factors and evaluated the predictive performance of four prognostic scoring systems, including Recursive Partitioning Analysis (RPA), Basic Score for Brain Metastases (BSBM), DS-GPA, and Lung cancer–associated molecular GPA (Lung-molGPA). Toriduka et al. (6) confirmed the validity of Lung-molGPA to estimate the median survival of LCBM patients who received their first course of radiotherapy. Study (7) externally validated the updated Lung-molGPA system and demonstrated that the Lung-molGPA could serve as a valuable tool for counseling of NSCLC patients with BM.

Overall, the traditional prognostic scores such as GPA, DS-GPA, and Lung-molGPA remain clinically useful for predicting survival in LCBM patients. Among these scores, Lung-molGPA generally provides better prediction by incorporating molecular biomarkers. However, these approaches are based on predefined prognostic factors and may not fully capture the complexity of patient outcomes.

### 3.2 RQ2: What are some existing models for predicting survival in LCBM patients?

Recent studies have proposed multiple data-driven approaches for survival prediction in LCBM patients, which can be broadly categorized into radiomics-based models, ML survival models, and DL frameworks.

Radiomics-based methods extract quantitative imaging biomarkers from MRI scans and utilize them for prognostic modeling. For example, Deng et al. (8) developed an interpretable prognostic model using Logistic Regression (LR) by combining MRI-based radiomic features with RNA sequencing data. The study utilized T1/T2 MRIs from multiple hospitals and identified four key radiomic biomarkers and highlighted the potential of combining radiomic and RNA sequencing data for improved survival prediction.

ML survival models have also demonstrated promising performance. Xie et al. (9) evaluated eight algorithms, including LR, Support Vector Machine (SVM), Random Forest (RF), and XGBoost (XGB). After identifying independent prognostic factors through Cox regression analysis, they developed an XGBoost-Surv framework to handle survival data and provide personalized survival curves. Luo et al. (10) developed prognostic models for OS prediction in SCLC patients with BM using Cox regression and American Joint Committee on Cancer (AJCC) staging as clinical baselines, alongside four ML algorithms, including Random Survival Forest (RSF), XGB, Elastic Net (E-Net), Artificial Neural Network (ANN). They found that the RSF model achieved the best performance. Likewise, Liu et al. (11) focused on developing a prognosis model for NSCLC patients with BM and evaluated the performances of four models, including RF, LR, SVM, and K-Nearest Neighbor (KNN), where the RF model outperformed all the other models.

DL approaches further extend predictive capabilities by automatically learning high-dimensional feature representations. The study (12) developed a DeepSurv (DL-based) model to predict OS and individualized risk-of-death periods by integrating MRI radiomic features, clinical data, and EGFR mutation status. The model was compared with lung-molGPA, which achieved a C-index of 0.66 and was limited by its inability to provide time-dependent survival predictions or individualized survival curves.

Overall, the relative strengths of radiomics, ML, and DL models differ. Radiomics models usually provide biologically relevant imaging biomarkers, ML models demonstrate strong predictive performance with moderate interpretability, and DL models are capable of learning complex feature representations.

### 3.3 RQ3: Does multimodal data integration improve survival prediction performance compared to unimodal models?

Multiple studies suggest that integrating imaging and clinical data may improve survival prediction in LCBM patients compared with unimodal approaches. Wang et al. (13) compared five survival models using different combinations of clinical features, CT-derived radiomics from the primary lung tumor, and brain MRI radiomics (T1+T1c) to predict progression-free survival (PFS). Their DL-based CoxCC model, which integrated BM radiomics, clinical features, and a prognostic index, achieved the highest time-dependent AUC (t-AUC) and consistently outperformed established scoring systems, including RPA, DS-GPA, lung-molGPA, and TNM staging (all p *<* 0.001). Study (14) demonstrated that model combining MRI radiomic features with clinical information yields higher predictive performance than those relying solely on either imaging or clinical data. Qi et al. (15) reported that combined clinical–radiomics nomograms consistently achieved higher Cindex values than either clinical-only or radiomics-only models for predicting short-term treatment response and intracranial progression-free survival (iPFS) in patients with EGFR-mutated lung adenocarcinoma and BM receiving EGFR-TKI therapy. Similar findings were reported in another study (16), which evaluated a comparable clinical-radiomics approach in a cohort of patients treated with third-generation EGFR-TKIs.

Overall, these studies indicate that multimodal models may outperform unimodal approaches. Nevertheless, differences in imaging modalities, prognostic factors, outcome definitions, and validation strategies across studies may limit direct comparison and remain a challenge for clinical implementation.

**Table 1** presents representative studies on survival prediction in LCBM patients and shows the methods used in recent research.

**Table 1:** Summary of survival prediction in lung cancer patients with brain metastasis studies.

Citation	Dataset	Method	Result				Limitations
			AUC	C-index	Statistical significance		
Sperduto et al (2022) (2)	4,183 patients from 20 institutions in three countries	Kaplan-Meier to estimate survival curves; Multiple Cox regression analysis for OS	NA	NA	Identified PD-L1 as a new significant prognostic factor for NSCLC adenocarcinoma (p < 0.01)		Retrospective design with inherent selection bias; lack of a standardized assay for PDL1 across all institutions; no data on discordance between primary tumor and BM molecular profiles
Sedef et al. (2023) (3)	95 NSCLC BM patients from two oncology centers in Turkey	Hospital-based case-series	NA	NA	p = 0.001 (among low, intermediate and high risk groups)		Retrospective study with unpredictable biases; evaluation did not include molecular subtypes
Schröder et al. (2023) (4)	137 NSCLC adenocarcinoma patients with BM	Kaplan Meier method to calculate OS	NA	NA	Chi-Square = 34.013, p < 0.001		Retrospective nature and limited amount of patients across certain subgroups
Citation	Dataset	Method	Result				Limitations
			AUC	C-index		Statistical Significance	
Miao et al. (2025) (5)	260 EGFRmutant lung AC patients who developed BM after receiving radical radiotherapy and chemotherapy	Evaluation of RPA, DS-GPA, BSBM, and Lung-molGPA systems; survival analysis using Kaplan-Meier and log-rank test	Lung-mol GPA: 0.875 (1-year OS), 0.876 (2-year OS) and 0.891 (3-year OS)	NA		Lung-mol GPA outperformed the other three systems (p < 0.05)	Retrospective and singlecenter study; lack of systematic data on patientreported outcomes, neurocognitive function, and treatmentrelated adverse events;
Toriduka et al. (2024) (6)	339 NSCLC patients with BM who received their first course of radiotherapy; subgroup of 65 patients received second-course radiotherapy	Validation of factor scores in the LungmolGPA system for adenocarcinoma and nonadenocarcinoma; validation of Lung-molGPA for survival prediction during first course of radiotherapy and secondcourse radiotherapy	NA	First-course radiotherapy: 0.65 (adenocarcinoma), 0.69 (non-adenocarcinoma); Second-course radiotherapy: 0.62 (adenocarcinoma), 0.74 (non-adenocarcinoma)		First-course radiotherapy: p<0.001 (adenocarcinoma), p<0.001 (non-adenocarcinoma); Secondcourse radiotherapy: p = 0.034 (adenocarcinoma), p = 0.022 (non-adenocarcinoma)	Retrospective and singleinstitution study; inclusion of only patients receiving first and second-course radiotherapy; lack of PDL1 expression data; old patient selection period
Crouzen et al. (2024) (7)	241 patients with BM from NSCLC adenocarcinoma treated with stereotactic radiotherapy	LungmolGPA score calculation; Kaplan–Meier for survival analysis	NA	0.77 (one year after diagnosis) and 0.71 (two years after diagnosis)		NA	Relatively small external cohort; limited to patients treated with best supportive care
Deng et al. (2025) (8)	41 patients from Xiangya Hospital and 86 patients from Yale New Haven Hospital	Univariate LR, Cox regression model for survival analysis and comparison; genomic and immune microenvironment validation (RNA-seq, GSEA, CIBERSORT, EcoTyper)	0.96 (training), 0.84 (test)	0.89 (training), 0.78 (test)		p = 0.01 (between high and low risk group)	Relatively small sample size for combined MRI/sequencing subset; Yale dataset lacked treatment and driver mutation details; 36month follow-up limitation
Xie et al. (2025) (9)	SEER database (n = 2624) and Harbin Medical University Cancer Hospital (n = 362)	Univariate and multivariate Cox regression–based prognostic factor selection, comparison of eight ML models, SHAP interpretability	XGBoostSurv model: 0.731 (training), 0.705 (test)	0.653 (training), 0.634 (test)		Lymph node metastasis significantly affected prognosis (p < 0.001)	Geographical or demographic limits; lack of immunotherapy or targeted therapy data; SEER database lacks details on disease recurrence
Luo et al. (2026) (10)	2,392 SCLC patients with BM; external validation cohort of 85 patients	Developed prognostic models using Cox proportional hazards model, AJCC staging, and four ML alogrithms - RSF, XGB, Enet, ANN	RSF modeltraining: 0.738 (1year), 0.809 (2-year); internal validation: 0.718 (1year),0.748 (2-year); external validation: 0.686 (1year),0.802 (2-year)	NA		NA	Retrospective study with potential selection bias; lack of detailed clinical information in the database; limited generalizability
Jingxin et al. (2025) (11)	3,171 Brainmetastasized non-small cell lung cancer (BM-NSCLC) patients from SEER database	Kaplan–Meier analysis for OS and cancer-specific survival (CSS); univariate and multivariate Cox regression for prognostic factor identification; development and evaluation of RF, LR, SVM, and KNN models;	RF modelvalidation: 0.89 (1year OS)	NA		NA	Lack of treatment information; insufficient diagnosis description; retrospective study with selection bias
Liao et al. (2023) (12)	237 NSCLC patients treated with Gamma Knife Radiosurgery (GKRS) at Taipei Veteran General Hospital	Univariate Cox (continuous variables)/chi-square (categorical variables) + sequential forward selection (SFS) feature selection; Applied DeepSurv model and statistical validation	DeepSurv model: 0.82 (3 months), 0.80 (6 months), 0.84 (12 months), 0.92 (24 months)	0.75		Differences between two risk groups (p < 0.015); DeepSurv outperformed lung- molGPA (p < 0.001)	Singleinstitution retrospective data; semiautomatic segmentation; wild-type EGFR patients demonstrate poorer outcomes
Wang et al. (2024) (13)	72 NSCLC patients with brain metastasis from Taipei Veterans General Hospital	Three-stage feature selection (univariate Cox proportional regression, Variance inflation factors, multivariate Cox proportional regression), CoxCC model was compared with other five models	CoxCC model: 0.88 (3 months), 0.73 (6 months), 0.92 (9 months) and 0.90 (12 months) for PFS prediction	0.75		p < 0.001 (between the high and low risk groups)	Single-center retrospective cohort; selection bias; exclusion of third-line TKIs; small dataset
Chen et al. (2021) (14)	110 NSCLC patients with BM, including 75 EGFR, 26 ALK, and 15 KRAS mutationpositive	Radiomic feature extraction; radiomic score construction via multivariate Cox regression; predictive model development for survival duration	0.977 (EGFR), 0.905 (ALK), 0.947 (KRAS)	NA		NA	Retrospective single-institution study; modest sample size; mutation status inferred from primary NSCLC; all confounding factors not evaluated
Qi et al. (2024) (15)	117 EGFRmutant NSCLC patients with BM who received EGFR-TKI treatment	MRI radiomics feature extraction; clinical risk factor screening; development of clinical (C), radiomics (R), and combined (C+R) nomograms	NA	combined C+R nomograms: short-term efficacy- 0.860 (training), 0.843 (validation); iPFS model- 0.837 (training), 0.850 (validation)		NA	Retrospective study with selection bias; small sample size; singlecenter study; third-generation EGFR-TKI cases excluded
Qi et al. (2024) (16)	194 EGFRmutated lung adenocarcinoma patients with BM treated with thirdgeneration EGFR-TKIs	MRI radiomics feature extraction, clinical risk factor screening; construction of Clinical (C), Radiomics (R), and combined C+R nomograms;	combined C+R nomograms: short-term efficacy- 0.858 (training); 0.804 (validation); iPFS- 0.895 (training); 0.936 (validation)	combined C+R nomograms: short-term efficacy- 0.867 (training); 0.803 (validation); iPFS- 0.901 (training); 0.753 (validation)		combined C+R nomograms: short-term efficacy- p < 0.001 (training); p = 0.045 (validation); iPFS- p < 0.0001 (training); p = 0.0059 (validation)	Single-center study with no validation; retrospective design with selection bias and confounding factors; multistep feature selection; variability in efficacy among three third-generation EGFR-TKIs;

OS, Overall Survival; PFS, Progression-Free Survival; iPFS, Intracranial Progression-Free Survival; AUC, Area Under the Curve; C-index, Concordance Index; NA, Not Available.

## 4 Critical analysis and future research directions

Recent ML and DL approaches have demonstrated strong performance; however, several methodological and clinical issues remain to be addressed. Many studies are retrospective and based on single-center cohorts with relatively small datasets, increasing the risk of overfitting. Although several models achieved high AUC or C-index values, they have not been widely validated on large, independent datasets from multiple centers. Moreover, few studies report calibration performance or decision-curve analysis, which raises concerns regarding model reliability, generalizability, and clinical applicability in real-world settings. Another challenge is heterogeneity among patient groups and treatment settings. Differences in reported endpoints (OS, PFS, iPFS) across studies limit direct comparison and benchmarking of predictive models. Variations in treatment periods, systemic therapy exposure, molecular subtype composition, radiotherapy approaches, and use of targeted therapies further complicate cross-study evaluations. Although multimodal AI-based models showed promising performance compared to unimodal approaches, it remains unclear whether these improvements translate into meaningful clinical benefits. In addition, these improvements in predictive accuracy require more extensive datasets, segmentation reproducibility, interpretability, and greater computational burden, which may limit their routine use in clinical practice.

This mini-review provides a focused and clinically relevant synthesis of survival prediction approaches. It specifically concentrates on LCBM patients rather than broader lung cancer cohorts, with particular emphasis on survival prediction endpoints (OS, PFS, iPFS). In addition, it discusses conventional prognostic tools alongside emerging AI-driven multimodal approaches. By integrating these perspectives, the review highlights not only recent methodological developments but also their translational relevance, clinical applicability, and current limitations in real-world clinical decision-making.

Therefore, future studies should utilize multi-institutional and multi-ethnic datasets to ensure generalizability and reduce overfitting in single-center populations. One potential research direction is the development of multimodal models integrating histopathological images, which may improve predictive performance. Furthermore, AI-based models should be compared with established prognostic tools in the same patient population to determine whether they provide incremental clinical value beyond existing approaches. Finally, explainable AI techniques can be embedded in model design to maintain clinician trust and facilitate adoption in routine practice.

## 5 Conclusion

Survival prediction in LCBM patients remains a clinically challenging and evolving area. The reviewed literature demonstrates that traditional scoring systems and unimodal approaches play an important role in survival prediction. However, these methods are limited in capturing the full biological and clinical complexity of these patients. Recent research suggests that multimodal integration may improve predictive performance. Nonetheless, these approaches have various limitations that must be addressed in future research.
